# Well-being and quality of life in people with disabilities practicing sports, athletes with disabilities, and para-athletes: Insights from a critical review of the literature

**DOI:** 10.3389/fpsyg.2023.1071656

**Published:** 2023-02-08

**Authors:** Luca Puce, Patrick Mbah Okwen, Mirabel Nain Yuh, Gloria Akah Ndum Okwen, Rigobert Hanny Pambe Miong, Jude Dzevela Kong, Nicola Luigi Bragazzi

**Affiliations:** ^1^Department of Neuroscience, Rehabilitation, Ophthalmology, Genetics, Maternal and Child Health (DINOGMI), University of Genoa, Genoa, Italy; ^2^Effective Basic Services (eBASE), Bamenda, Cameroon; ^3^Laboratory for Industrial and Applied Mathematics (LIAM), Department of Mathematics and Statistics, York University, Toronto, ON, Canada; ^4^Africa-Canada Artificial Intelligence and Data Innovation Consortium (ACADIC), Department of Mathematics and Statistics, Faculty of Science, York University, Toronto, ON, Canada

**Keywords:** global well-being, quality of life, hedonic well-being, eudaimonic well-being, sports-related well-being, disabled athletes and para-athletes, critical review

## Abstract

Global well-being (GWB) is a complex, multi-dimensional, and multi-faceted construct that can be explored from two different, but often overlapping, complementary perspectives: the subjective and the objective ones. The subjective perspective, in turn, is comprised of two dimensions: namely, the hedonic and the eudaimonic standpoints. Within the former dimension, researchers have developed the concept of subjective hedonic well-being (SHWB), whereas, within the latter, they have built the framework of psychological and social well-being (PSWB). Disabled people have poorer well-being due to their pathology and may more frequently suffer from anxiety and depressive disorders than their able-bodied counterparts. Sports participation is an essential way to cope with disability. On the other hand, compared with their able-bodied peers, athletes with disabilities and para-athletes undergo a unique series of stressors. Little is known in terms of hedonic and eudaimonic well-being and quality of life in this specific population. Here, we review the literature, with an emphasis on the current state-of-art and gaps in knowledge that need to be addressed by future research. High-quality, large-scale investigations are needed to have a better understanding of the self-perceived (hedonic) and objective (eudaimonic) well-being and quality of life of disabled people practicing sports, athletes with disabilities, and para-athletes.

## Well-being and quality of life

Global well-being (GWB) is a complex, multi-dimensional, and multi-faceted construct that can be explored from two different, but often overlapping, complementary perspectives: the subjective and the objective ones. The subjective perspective, in turn, is comprised of two dimensions: namely, the hedonic and the eudaimonic standpoints (Ryan and Deci, 2001; Ryff et al., 2021). Within the former dimension, researchers have developed the concept of subjective hedonic well-being (SHWB; Diener, 1984; Busseri and Sadava, 2011), whereas, within the latter, they have built the framework of psychological and social well-being (PSWB; Waterman, 1993; Keyes, 1998; Ryff, 2014).

SHWB relates to how individuals experience and rate different aspects of their lives and can be defined as “a broad category of phenomena that includes people’s emotional responses, domain satisfactions, and global judgments of life satisfaction” (Diener et al., 1999). This construct is generally employed to quantitatively evaluate mental health and happiness, and it has been found to be a major predictor of individual wellness, health, and longevity (Sears et al., 2014). SHWB can be conceived as “tripartite,” there is to say, consisting of three broad components: namely (i) life satisfaction (long-term rating of satisfaction overall or domain-specific, referring to the workplace, partners, friends/colleagues, children, etc.); (ii) positive affect; and (iii) negative affect (Busseri and Sadava, 2011; Ryff et al., 2021). Happiness is conceived as the balance between positive and negative affect (Diener et al., 2005). Among the different existing instruments (Cooke et al., 2016), SHWB can be measured using a widespread and well-documented survey index, namely the “Psychological General Well-Being Index” (PGWBI; Dupuy, 1984), which provides an assessment of self-perceived psychological well-being in terms of different domains, including (i) depressed mood; (ii) anxiety; (iii) vitality; (iv) positive well-being; (v) self-control; and (vi) general health.

PSWB consists of psychological well-being (PWB) and social well-being (SoWB). The former can be understood according to the six-factor model, which sees PWB as a construct consisting of six components: namely (i) awareness and acceptance of personal limitations (self-acceptance); (ii) cultivating positive connections, and meaningful relationships with others; (iii) being self-determining, and setting goals based on personal convictions and standards (autonomy); (iv) navigating life’s circumstances (environmental mastery); (v) attributing meaning and direction to life (purpose in life); and (vi) being welcoming to new experiences, continuously developing and improving oneself over time (personal growth). These components are all essential and mutually influence each other (Ryff, 1989; Ryff, 2014). Similarly, SoWB is comprised of the following dimensions: (i) social acceptance; (ii) social actualization; (iii) social contribution; (iv) social coherence; and (v) social integration (Lundqvist and Sandin, 2014; Joshanloo, 2022).

Despite being conceptually different, at least partially, the two models of well-being (hedonic and eudaimonic) are overlapping, with prominent theorists (such as Aristotle, Jung, Maslow, Allport, Rogers, Erikson, Frankl, Jahoda, Neugarten, or Bühler) having contributed to the development of both (Ryff, 1989; Ryff and Singer, 2008; Ryff, 2016).

These two concepts parallel the dichotomy introduced in the field of behavioral economics and applied psychology by Daniel Kahneman: (i) “experienced well-being,” which corresponds to hedonic well-being (as previously said, a dynamic balance between positive affect, pleasure, and happiness, and negative affect, distress, or misery); and (ii) “evaluative wellbeing,” which corresponds to eudaimonic well-being (that is to say, autonomy, personal growth, and meaning/purpose in life; Kahneman, 1999; Salvador-Carulla et al., 2014).

The concept of objective well-being defines well-being in terms of quality of life indicators, as “the list of goods that are necessary for a good life” (Bohnke and Kohler, 2008) including material resources (like income, food, or housing) and social attributes (such as education, health, “political voice,” or social capital, like family, friendship and social networks and connections, and social inclusion), among others (Western and Tomaszewski, 2016).

The objectivist approach to well-being has been mostly pioneered by Amartya Sen, with his work in welfare economics (Sen, 1973). Another prominent theorist and scholar of objective well-being is Martha Nussbaum (Anand et al., 2004). Altogether, their contributions are known as the Sen–Nussbaum approach to well-being. Objective well-being is also, sometimes, called “contextual well-being.”

**Figure 1:**
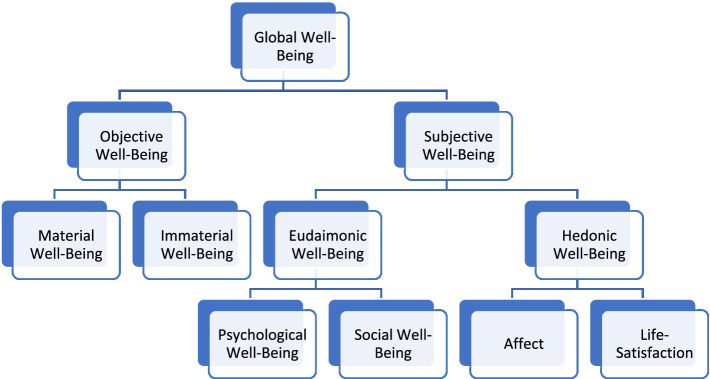
A summary of the various dimensions of well-being.

The various dimensions of global well-being are summarized in Figure 1.

These various concepts of well-being have been recently adapted and translated, as well as integrated, into the sports world (Lundqvist and Sandin, 2014). Well-being, as experienced by athletes, especially elite ones, is particularly rich, complex, and nuanced, depending also on the specific context that surrounds the athlete (Lundqvist, 2011; Lundqvist and Sandin, 2014). The sports arena is, indeed, challenging and rewarding at the same time, as it provides venues to explore new opportunities, experience success as well as failure, and interact and connect with peers (Mack et al., 2012). On the other hand, athletes have to cope with heavy training schedules, psychological challenges, like internal and external pressures, various transition phases, and logistic-organizational stressors, as well as injuries, and performance plateau (Lundqvist and Sandin, 2014). Global and sports-related well-being can be conceived as “an interplay of satisfaction with life, sport experiences and perceived health combined with experienced enjoyment and happiness in both ordinary life and sport” (Lundqvist and Sandin, 2014).

According to Lundqvist (2011), global and sports-related well-being consists of a hedonic component [SWB in sport (SWB-S)], and of a eudaimonic component. SWB-S consists of sports satisfaction, and sports-related affect, while PWB in sport (PWB-S) is comprised of (i) self-acceptance as an athlete, (ii) positive relation to the coach and teammates, (iii) autonomy in sports practice, (iv) sports environmental mastery, (v) purpose in sport, and (vi) personal growth as an athlete. Finally, SoWB in sport (SoWB-S) consists of (i) social acceptance in sport, (ii) social actualization through sport, (iii) social contribution to sport, (iv) social coherence in sport, and (v) social integration in sport.

Related to well-being, there are other constructs, like the quality of life (Prutkin and Feinstein, 2002), health-related quality of life (HRQoL), happiness, human functioning, and health-related human functioning (HRHF; Salvador-Carulla et al., 2014), which can be regarded as (sometimes overlapping, sometimes different and complementary) subcategories of well-being (Salvador-Carulla et al., 2014). The former construct can be defined as “a person’s perception of his/her position in life within the context of the culture and value systems in which he/she lives and in relation to his/her goals, expectations, standards, and concerns” (The WHOQOL Group, 1994). The latter constructs (namely, human functioning, and HRHF) are relevant when it comes to the scholarly investigation of disability.

## Disability

Disability can be defined as “a difficulty in functioning at the body, person or societal level, in one or more domains, as experienced by an individual with a health condition in interaction with contextual factors” (Raggi et al., 2010). According to the World Health Organization (WHO), a person with a disability can be defined as a person having “a problem in body function or structure, an activity limitation,” and/or “a difficulty in executing a task or action; with a participation restriction.” People with disabilities represent a large portion of the general population, currently being more than 1 billion people worldwide. They have to cope with (either structural or perceived) obstacles and barriers that hinder their full participation in society and engagement with daily activities.

Currently, there is disagreement about the most respectful and appropriate way to refer to individuals with disabilities: “person-first language” (which focuses on the person rather than the disability), or “identity-first language.” Here, we want to acknowledge that, while the first option has the benefit of emphasizing the person’s individuality with the intention of reducing disability-related discrimination and stereotypes, on the other hand, its use may sound “awkward” and “unconventional” (Taboas et al., 2022). Paradoxically and unintentionally, its use could result in drawing “attention to the disability” (Taboas et al., 2022). Also, the disability community is beginning to “support the use of identity-first language that embraces all aspects of one’s identity” (Taboas et al., 2022), different from professionals who work in the disability community (Taboas et al., 2022). However, some survey-based studies seem to suggest that “both types of language are preferred by different groups of … [disability] stakeholder groups” (Taboas et al., 2022). Since language is highly dynamic and constantly under flux and the choice of “person-first language” or “identity-first language” reflects the evolution of language, culture, and society (Krista et al., 2022), in this critical review, we will use a mix and a variety of language, alternating between “person-first language” and “identity-first language,” choosing to use terms flexibly throughout our work. In doing so, we follow the suggestions of Dunn and Andrews (2015). We are aware of this choice and we clearly state it as a “reflexive research practice” (Krista et al., 2022). In this way, the reader can have a clear understanding of the choices and decisions, we have made while conducting the research and drafting our manuscript. Also, we state that we stand and will always stand with the members of the disability community and that we do not have any demeaning or derogatory attitude toward them.

A fair, just society should ensure the observation and application of the principles of gender, equity, inclusion, and diversity (GEID). People with disabilities have the right to access school, workplace, and justice, receive healthcare provisions and take part in cultural and sports activities, as stated by the United Nations (UN) Human Rights Office of the High Commissioner. However, despite being apparently protected by the law, the voices of subjects with disability are generally unheard and their needs are often unmet. In the last decade, the 2008 “UN Convention on the Rights of Persons with Disabilities” has reiterated the societal onus to ensure people with disabilities, as well as other vulnerable and marginalized populations, dignity, respect, and human rights. The inclusion of diverse athletes, like those with a disability, has been growing in the last years, with paralympic events attracting a significantly increased portion of para-athletes, since the first sports event (“Silent Games”) took place in 1924, in Paris (France), involving 148 disabled athletes from a few European countries. Initially conceived as a rehabilitation sport, based on the vision of Dr. Ludwig Guttmann (1899–1980; Chun et al., 2021), inclusive sport has gradually shifted to recreational and competitive sport. In 1960, the first edition of the Paralympic Games was organized. Despite this, athletes with disability remain significantly sidelined in the sports community and in the coverage by mass and social media (Wolbring and Martin, 2018). In the existing scholarly literature, athletes with a disability are dramatically under-represented with respect to their able-bodied counterparts, with a significant dearth of data and available evidence concerning their well-being and quality of life (Macdougall et al., 2015), determinants of fatigue and performance outcomes, as well as optimal training programs and strategies, and rehabilitation protocols.

Generally, people with disabilities report poorer well-being due to their health status and underlying conditions, and may more frequently suffer from anxiety and depressive disorders than their able-bodied counterparts (Krahn et al., 2015; Tough et al., 2017), even though they can develop particular skills and strategies in order to face adverse situations—this is known as the “disability paradox” (Albrecht and Devlieger, 1999), even if it has been questioned and challenged by some scholars (Koch, 2000). Being engaged in regular, structured physical activity, like sports participation, is an essential way to adapt to and cope with disability (Shephard, 1991; Ascione et al., 2018; Kiuppis, 2018; Puce et al., 2019; Maugeri et al., 2020). On the other hand, compared with their able-bodied peers, disabled athletes known also as para-athletes undergo a unique series of stressors that deeply influence the process of forming a new identity (Brewer et al., 1994), such as physical access, communication, or economic-financial barriers, discriminating, and demeaning attitudes, and unprofessional coaching (Iezzoni, 2009; Jefferies et al., 2012). If practicing sports can result in improved inclusion, and, therefore, enhanced self-acceptance as well as social acceptance (Trigueros et al., 2021), less is known in terms of well-being, both from a subjective and objective perspective, in this specific population.

## Well-being and quality of life in people with disabilities practicing sports, athletes with disabilities, and para-athletes

A systematic review of the literature (Macdougall et al., 2015) retrieved 12 studies comparing the well-being of Para and Olympic sports athletes. However, the authors found that there were insufficient data to conduct a meta-analysis for the dimension of SWB−life satisfaction or long-term affect. Moreover, the effect sizes from individual studies were contrasting, both in terms of magnitude and direction. While two studies (Horvat et al., 1989; Wisniowska et al., 2012) reported statistically significant differences in favor of Olympic sports athletes for life satisfaction, total mood-disturbance differences, fatigue, and depression, one study (Pensgaard et al., 1999) reported opposite findings, by computing significant differences in favor of para-athletes for satisfaction with effort and results from a major competition. Finally, two studies (Horvat et al., 1989; White and Croce, 1992) could not report any significant differences between the two athletic populations for long-term affect across anger, anxiety, confusion, tension, or vigor. Besides such conflicting findings, even fewer studies exist comparing para-athletes and disabled subjects non-practicing competitive para-sports.

As such, there seems to be little evidence of the psychological benefits of competitive sports for disabled individuals, probably due to the paucity of studies addressing this topic. Moreover, the existing scholarly research is limited to specific disabilities, para-sports disciplines, settings, and geographic contexts, with samples generally consisting of a limited number of participants. Furthermore, there is a marked lack of comparative data exploring the differences in well-being between para-athletes and individuals with disabilities who do not play competitive sports. Therefore, given this dearth of information, this review study was conducted to fill in this gap of knowledge.

Available research (either observational or interventional) conducted on able-bodied athletes and the general population has emphasized the value of different forms of physical activity, either unstructured or structured (including exercise, and sport), in terms of the promotion and enhancement of various components of well-being and physical self-perception, with a “multiplier effect,” with engagement improving general health and well-being, which, in turn, encourages further sports participation, with subsequent further enhancements in general health and well-being, resulting in a virtuous circle (Downward et al., 2018). Improvements in both hedonic and eudaimonic well-being were found. For instance, Edwards et al. (2004) explored the relationship between sports involving diverse types of regular exercise, such as hockey and health club activities (team and individual sports involving aerobic and resistance exercise, respectively), and mental and physical health. The authors measured eudaimonic well-being, by utilizing Ryff’s conceptual framework. The authors recruited and compared 60 university hockey players, 27 health club members, and 111 non-exercising students. The latter population was found to display less well-being and physical self-perception. Specifically focusing on SHWB, Wilson et al. (2022) quantitatively assessed the correlation between sports participation and well-being in cohorts of adolescents (aged 11–17 years), in New Zealand. Hedonic well-being was assessed utilizing a single-item graded on a 10-point Likert scale ranging from 1 (“very unhappy”) to 10 (“very happy”), following the “Organisation for Economic Co-operation and Development” (OECD) guidelines on measuring SHWB. Better hedonic well-being was found to be associated with participation in any sport vs. none. Of note, well-being was not associated with participation in physical education or *solo* sport. During the still ongoing “Coronavirus Disease 2019” (COVID-19) pandemic, sports students exhibited higher levels of SHWB (increased positive affect and reduced negative affect), when compared with music students (Habe et al., 2021). Several parameters were identified mediating the link between sports participation and SHWB, including age, sex/gender, income, relationship status, intensity, and duration of physical activity, among others (Ruseski et al., 2014; Wicker and Frick, 2015; Zhang et al., 2022). Overall, physical activity was found to be related to positive affect, but unrelated to negative affect, enhancing SHWB, with effects consistently shown across all age groups and a variety of settings (individual vs. team sports, light vs. moderate and hard intensity, aerobic vs. anaerobic and mixed exercise), and prior fitness levels (Buecker et al., 2021).

Specifically concerning competitive sports, some studies (Saw et al., 2016; Watson et al., 2017; Abbott et al., 2018; Watson and Brickson, 2018, 2019) identified some associations between SHWB and sports-related parameters, like training load, training-induced stress (Saw et al., 2016; Watson et al., 2017; Watson and Brickson, 2018, 2019), match location, match result, and the quality of the opposition during a soccer match (Abbott et al., 2018), as well as social identification with college sports teams (Graupensperger et al., 2020). In general, the authors deployed in-house developed questionnaires, with a few studies using reliable instruments complemented by the use of objective measures.

Less is known about the impact of sports participation on well-being among the disabled population, even though a growing body of scholarly research has shown that practicing sports at a competitive level such as Paralympic sports, directly and indirectly (through the related emotional, motivational, and social characteristics that characterize the sports environment), could make a greater contribution to the SHWB and PSWB of individuals with disabilities, helping them grow and cope with the challenges of life, favoring the acceptance of one’s health status, the assumption of responsibilities, and the achievement of personal goals (Puce et al., 2017).

Previous surveys specifically focusing on the perceived psychological and emotional well-being of para-athletes compared with disabled people who did not practice competitive sports have shown greater well-being of the former population, underlining the strength of competitive sports, which are able to act on different areas such as (i) the emotional sphere, through the experience of achieving a predetermined goal; (ii) the motivational sphere, through the possibility of competing fairly with opponents having the same degree of functionality; (iii) the social sphere, through the establishment of lasting, meaningful interpersonal relationships within the team; and (iv) the physical sphere, through the maximization of residual motor capacity and the development of new abilities.

For instance, para-sports such as para-swimming have been shown to be useful for facilitating self and social acceptance, for the development of identity and a sense of normalization (Pack et al., 2017), improving the quality of life, reducing anxiety, and increasing self-esteem (Vita et al., 2016).

These findings are comparable to previous surveys of wheelchair sports (like basketball, tennis, and rugby) competition participants. The researchers found that para-athletes have lower rates of depression, tension, anger, and confusion, as well as an increase in life satisfaction (Paulsen et al., 1990; Fiorilli et al., 2013; Nagata, 2014) and more positive perceptions of one’s health and well-being than non-para-sports participants (Greenwood et al., 1990; Campbell and Jones, 1994; Martin et al., 2011; Litchke et al., 2012).

A recently published survey (Mira et al., 2022) studied 31 of the 33 athletes of the Portuguese Paralympic team (aged 34.45 ± 11.7 years, 21 men and 10 women), participating in several para-sports disciplines (namely, para-athletics, para-badminton, boccia, para-canoe, para-cycling, equestrian, judo, and para-swimming). The findings highlighted high values of life satisfaction, high positive affect, and low negative affect levels. Moreover, the authors were able to report high levels of resilience and social support.

However, due to the extremely competitive sports environment para-athletes can experience several sport-specific and disability-specific stressors that are potentially detrimental to personal well-being (Macdougall et al., 2016). For example, the training methodologies in terms of volumes, intensity, and recoveries of para-athletes are very similar to those of normal athletes, even if each type of impairment may respond differently to the training load, and this can lead to overtraining, burnout, pain, and injuries (Puce et al., 2018). Furthermore, frequent travel, often difficult from a logistical point of view, can lead to greater psychological stress levels, reduction in the quantity (hours) and quality of sleep, and eating disorders.

Also, there is the possibility that a para-athlete will be assigned to an incorrect functional para-sports class, this could cause frustration, poor sport-related satisfaction, and, in some cases, retirement from competitions (Swartz et al., 2019).

Finally, there is also evidence that participation in competitive sports has an impact on the athletic identity of individuals with disabilities (Kokaridas et al., 2009; Pack et al., 2017). Perceiving oneself exclusively as an athlete implies not only positive aspects such as motivation, goal orientation, and sense of empowerment, but also negative aspects such as exclusivity (i.e., inability to identify with other roles) and negative affectivity (i.e., negative emotional responses to injury, retirement, or other sources; Martin et al., 1995, 1997).

Limitations of the overviewed studies include their cross-sectional study design, the use of either only self-report measures that may result in reporting and recalling bias, or objective measures, without exploring the subjective perspective of the participants. Several para-sports disciplines are not represented in the literature and some of those investigated may be under-represented. Moreover, the sample size of these studies is usually small. Further, several existing studies are not underpinned by a precise psychological theory/framework of well-being, and some of them fail to capture its multi-dimensional nature, using tools consisting of a single item or a few items, instead of employing a theoretically grounded, psychometrically sound and multi-faceted tool, specifically devised for disabled people and para-athletes. Also, indicators and scales have been developed and tested predominantly in the Global North, with populations mainly consisting of white, male university students. As such, the measures and indicators should not be assumed to be applicable to other populations. The disability community is heterogeneous, but its variety has not been sufficiently captured by the scholarly literature. More attention to GEID principles should be paid.

## Conclusions and future directions

This review study contributed to a better understanding of subjective and objective well-being and quality of life among people with disabilities practicing sports, athletes with disabilities, and para-athletes. However, future studies should elucidate the relationships between hedonic and eudaimonic well-being in this specific population, especially from a longitudinal (rather than cross-sectional) perspective. Future indications also include the investigation of the mechanistic pathways that can link practicing sports with well-being outcomes in the disabled population. These studies should adopt a more multi-dimensional perspective, attempting to disentangle the complexities underlying overlapping/complementary constructs such as well-being (GWB, hedonic/SHWB, eudaimonic/PSWB, and objective/contextual well-being), quality of life, HRQoL, human functioning, and HRHF. Particular effort should be paid avoiding to present disability through a medical model lens, with impairment as a medicalized defect of functioning (Smith et al., 2016; Bundon et al., 2022).

Currently, a comprehensive, conceptually and theoretically grounded, scholarly sound map/taxonomy of an array of health-related “meta-constructs” or “meta-categories” (wellbeing, health condition/health status, human functioning, disease/pathology, disability, etc.) is urgently needed (Cieza and Stucki, 2008; Salvador-Carulla and Gasca, 2010). A mapping/scoping exercise should be conducted to identify operational definitions of these identities/meta-identities, their conceptual hierarchy, and their granularity and complexities, in terms of the various (sub-)domains, (sub-)dimensions, and (sub-)facets (Salvador-Carulla et al., 2014). This should lead to a person-centered framework “ranging from ill-health/ill-being to good-health/well-being that incorporates all major aspects of well-being in its preliminary conceptual map: positive and negative polarity, condition status and functioning, experiences of health and contributors to ill and to good health” (Mezzich et al., 2010; Salvador-Carulla et al., 2014).

Truly inclusive health, disease, and disability ontologies are still lacking (Sefotho, 2021), with health and well-being usually understood as normative, rather than foundational concepts. There is also a lack of tools for assessing the well-being of persons with disabilities. A major step forward is represented by the “World Health Organization Quality of Life” (WHOQOL) disabilities module (WHOQOL-DIS) for people with physical and intellectual disabilities (Power et al., 2010). Moreover, these constructs can be complemented by the assessment of the so-called “objective well-being” and related constructs, such as human flourishing and capabilities (Nussbaum, 2006; Bloodworth et al., 2012). In the specific case of disabled subjects, objective scales include the Karnofsky index, which was introduced in the healthcare field to quantitatively assess the performance status of patients with malignancies and people with disabilities, the clinical indexes of “Activities of Daily Living” (ADL), and the “World Health Organization Disability Assessment Schedule 2.0” (WHODAS 2.0; Karnofsky and Burchenal, 1948; Katz et al., 1963; Ustün et al., 2010; Kostanjsek et al., 2011; Na and Streim, 2017).

This would advance our understanding of disability and would assist and inform the data-driven, evidence-based design and implementation of interventions aimed at improving and enhancing the well-being, quality of life, and functioning of disabled people (Ferrario and Guarino, 2009; Riddle, 2013). Understanding disability status and associated well-being can help policy- and decision-makers, as well as service providers, devise adequate, effective programs. Currently, only a few scales exist assessing GWB in disabled individuals from both a subjective and objective standpoint, including the “Integral Quality of Life Scale” (Verdugo et al., 2009), consisting of eight major domains (self-determination, rights, emotional well-being, social inclusion, personal development, interpersonal relationships, material well-being, and physical wellbeing), which, however, has been developed for and tested in persons with intellectual disabilities. To these domains, Davidson et al. (2017) have added the following: environment, family, recreation and leisure activities, and, safety/security. Finally, health-, well-being-, disability-related ontologies, and semantic maps can be “translated” and “adapted” to the sports arena, and connected with sports-related ontologies and semantic maps (Ramkumar and Poorna, 2017), to assist sports scientists and managers, instructors, and coaches in the development of adequate training strategies.

## Author contributions

LP, PO, MY, GA, RP, JK, and NB conceived and drafted the manuscript. All authors contributed to the article and approved the submitted version.

## Funding

This research was funded by Canada’s International Development Research Centre (IDRC) and the Swedish International Development Cooperation Agency (SIDA) (Grant No. 109559-001). NB and JK acknowledge support from IDRC (Grant No. 109981). JK acknowledges support from New Frontier in Research Fund-Exploratory (Grant No. NFRFE-2021-00879) and NSERC Discovery Grant (Grant No. RGPIN-2022-04559).

## Conflict of interest

PO, MY, GA, and RP were employed by Effective Basic Services (eBASE).

The remaining authors declare that the research was conducted in the absence of any commercial or financial relationships that could be construed as a potential conflict of interest.

## Publisher’s note

All claims expressed in this article are solely those of the authors and do not necessarily represent those of their affiliated organizations, or those of the publisher, the editors and the reviewers. Any product that may be evaluated in this article, or claim that may be made by its manufacturer, is not guaranteed or endorsed by the publisher.
